# Association of insulin resistance-related indicators with cardiovascular disease in Chinese people with different glycemic states

**DOI:** 10.3389/fendo.2025.1515559

**Published:** 2025-04-17

**Authors:** Zihao Zhang, Lan Tan

**Affiliations:** Department of Neurology, Qingdao Municipal Hospital, Qingdao University, Qingdao, China

**Keywords:** stroke, heart disease, insulin resistance, cardiovascular, glucose

## Abstract

**Background:**

This study compares the association of eight insulin resistance (IR)-related markers (triglyceride-glucose index (TyG), TyG-body mass index (TyG-BMI), TyG-waist circumference (TyG-WC), TyG-waist-to-height ratio (TyG-WHtR), triglycerides-to-high-density lipoprotein cholesterol ratio (TG/HDL), lipid accumulation product (LAP), visceral adiposity index (VAI), and estimated glucose disposal rate (eGDR)) with cardiovascular disease (CVD).

**Methods:**

Spearman’s coefficients were used for correlations between IR-related markers. Predictive capacities were evaluated using receiver operating characteristic (ROC) curve analysis, Akaike Information Criterion, and Bayesian Information Criterion were calculated. Multivariable-adjusted Cox regression models and restricted cubic spline (RCS) analysis were performed to explore associations between IR-related markers and CVD.

**Results:**

In Pearson correlation analysis, TyG-WC and TyG-WHtR had a correlation coefficient of 0.95, while TG/HDL ratio and VAI had a correlation coefficient of 0.97. Regarding predictive capacity across different glycemic states, eGDR showed the best performance among the 8 IR-related markers, particularly in predicting stroke. According to Cox regression analysis, with each unit increase in TyG, TyG-BMI, TyG-WC, and TyG-WHtR, the risk of heart disease increased by 24.1%, 0.4%, 0.1%, and 17.56%, respectively; and the risk of stroke increased by 69.3%, 0.6%, 0.2%, and 36.5%, respectively. Additionally, TG/HDL ratio, VAI, and LAP exhibited nonlinear associations with heart disease and stroke risk. For each unit increase in eGDR, the risks of heart disease and stroke decreased by 21% and 14.2%, respectively

**Conclusion:**

eGDR is the most effective marker for predicting CVD, especially stroke, across all glycemic states. Modified TyG indices provide better predictive value than TyG alone.

## Background

According to data from the World Health Organization, cardiovascular diseases (CVD) are the leading cause of death globally, responsible for approximately 17.9 million deaths annually, accounting for 32% of all deaths worldwide (1). Early identification and intervention for modifiable risk factors are crucial in mitigating the impact of CVD. Major risk factors for CVD include hypertension, high cholesterol, smoking, diabetes, obesity, and physical inactivity (2). Insulin resistance (IR) is a significant determinant of CVD, contributing to its development through mechanisms such as endothelial dysfunction, increased inflammatory responses and dyslipidemia (3).

Given the critical role of IR in CVD, it is essential to identify practical and reliable markers to assess IR and its associated risks. Unlike the expensive and time-consuming hyperinsulinemic-euglycemic clamp technique (HEIC), the triglyceride-glucose (TyG) index is simple, cost-effective, and has become a practical alternative for assessing IR (4). Combining the TyG index with other obesity indicators can enhance its predictive power for CVD (5, 6). For example, the TyG- body mass index (TyG-BMI) index is more accurate in identifying high-risk individuals compared to the TyG index alone. Additionally, other IR-related markers have shown significant advantages in predicting CVD risk. The TyG-waist circumference (TyG-WC) and TyG-waist-to-height ratio (TyG-WHtR) indices incorporate central obesity measures, providing a more comprehensive assessment of metabolic health (7). Similar to the TyG index, the triglycerides-to-high-density lipoprotein cholesterol (TG/HDL) ratio is a simple and easily obtainable marker that can serve as a surrogate for IR, which can reflect lipid metabolism and the associated cardiovascular risk more comprehensively (8). The Lipid Accumulation Product (LAP) index provides a comprehensive measure of lipid accumulation and its impact on cardiovascular health. LAP is particularly effective in capturing the relationship between visceral fat and cardiovascular risk, making it a valuable tool for predicting CVD (9). Similarly, the Visceral Adiposity Index (VAI) is instrumental in evaluating visceral fat distribution, especially abdominal fat, which is a well-established risk factor for CVD. By offering detailed insights into fat distribution, VAI enhances the ability to stratify and manage cardiovascular risk more precisely (10). The estimated glucose disposal rate (eGDR) offers a direct measure of insulin sensitivity and glucose metabolism efficiency. It is calculated using variables such as waist circumference, hypertension status, and HbA1c levels, making it a comprehensive marker of metabolic health and a strong predictor of CVD risk (11). Despite the research on individual markers, there is a lack of studies investigating the association of these eight IR-related markers with CVD.

Additionally, there is an association between IR-related markers and the severity of CVD depending on glycemic status (12). However, previous research has primarily focused on evaluating the relationship between IR-related markers and cardiovascular disease in either diabetic or non-diabetic populations. Prior studies have found a correlation between the TyG index and coronary artery disease (CAD), with this correlation being strongest in diabetic patients (13). The TyG-WC and TyG-WHtR have also demonstrated good predictive ability for cardiovascular risk in non-diabetic populations (14, 15). In contrast, eGDR has been found to be a reliable predictor of CVD in both diabetic and non-diabetic individuals (16, 17). Given these findings, there is a need to explore further the role of these markers across different glycemic states. Therefore, this study aims to investigate the association and predictive capacity of IR-related markers with CVD across different glycemic states using a nationally representative cohort.

## Methods

### Study population

The China Health and Retirement Longitudinal Study (CHARLS) is an ongoing nationally representative survey, which initiated its baseline survey in 2011 and has been updated every two to three years, with follow-ups conducted in 2013, 2015, 2018, and 2020 (18). We utilized data from the baseline and four follow-up waves. A total of 6009 participants were included in the analysis, all of whom had available physical examination data, blood data, demographic data, and other required information. The specific inclusion and exclusion criteria are detailed in **Supplementary Figure 1**.

### Insulin resistance-related markers and glycemic states

FBG and TG levels were analyzed using a Hitachi 7180 chemical analyzer (Hitachi, Tokyo, Japan). Height, weight, WC, systolic blood pressure (SBP) and diastolic blood pressure (DBP) were measured three times, and the average value was taken as the final measurement. Hypertension was assessed through a standardized question: “Have you ever been told by a doctor that you have been diagnosed with hypertension?” (19) For participants who did not report whether they had hypertension, SBP ≥140 mmHg or DBP ≥90 mmHg was also considered hypertensive. The calculation formulas for the 8 insulin resistance-related markers are shown in **Table 1** (10, 11, 20).

**Table 1:** Formulas of insulin resistance-related markers.

IR-related markers	Formulas
TyG index	TyG = ln[fasting triglycerides (mg/dL) × fasting glucose (mg/dL)/2]
TyG-BMI index	TyG-BMI = TyG × BMI (kg/m²)
TyG-WC index	TyG-WC = TyG × waist circumference (cm)
TyG-WHtR index	TyG-WHtR = TyG × waist circumference (cm)/height (cm)
TG/HDL ratio	TG/HDL = triglycerides (mg/dL)/high-density lipoprotein cholesterol (mg/dL)
LAP index	Male: LAP =(waist circumference (cm) - 65) × triglycerides (mmol/L)
	Female: LAP =(waist circumference (cm) - 58) × triglycerides (mmol/L)
VAI	Male: VAI = [WC (cm)/(39.68 + (1.88 × BMI))] × (TG (mmol/L)/1.03) × (1.31/HDL (mmol/L))
	Female: VAI = WC (cm)/(36.58 + (1.89 × BMI))] × (TG (mmol/L)/0.81) × (1.52/HDL(mmol/L))
eGDR index	eGDR = 21.158 − (0.09 ×WC(cm)) − (3.407 × hypertension (yes=1/no=0)) − (0.551 × HbA1c (%))

TyG, triglyceride-glucose index; BMI, body mass index; WC, waist circumference; WHtR, waist-to-height ratio; TG, triglyceride; HDL, high-density lipoprotein; LAP, lipid accumulation product; VAI, visceral adiposity index; eGDR, estimated glucose disposal rate; HbA1c: glycated hemoglobin.

Blood glucose classification was based on FBG, glycated hemoglobin (HbA1c), and diabetes history. Participants were categorized into three groups: normal glycemic levels (FBG <100 mg/dL and HbA1c <5.7%), prediabetes (FBG 100-125 mg/dL or HbA1c 5.7-6.4%), and diabetes (FBG ≥126 mg/dL or HbA1c ≥6.5% or a history of diagnosed diabetes) (21).

### Heart disease and stroke

The incidence of heart disease and stroke was determined based on participants’ self-reports, confirming that they had received a definite diagnosis from a doctor. New cases of heart disease and stroke were identified as participants who reported having heart disease or stroke during the follow-up period (22).

### Covariates

Covariates included sociodemographic factors, blood biomarkers, and diseases related to CVD. Sociodemographic factors included age, sex, education level, marital status, smoking status, and alcohol consumption. Blood biomarkers included total cholesterol, triglycerides, creatinine, blood urea nitrogen, uric acid, and C-reactive protein. Diseases related to cardiovascular disease included hypertension, diabetes, and kidney disease.

### Statistical analysis

Continuous variables are presented as mean ± standard deviation (SD), and categorical variables are presented as number (percentage). Participants were divided into three groups based on glycemic status. Kruskal-Wallis test and analysis of variance (ANOVA) were used to describe baseline characteristics, IR-related markers, blood indicators, and body measurements for continuous variables, while the Chi-square test was used for categorical variables.

The correlations between the IR-related markers were expressed using Pearson’s coefficients. The predictive capacities of the IR-related markers for heart disease and stroke in the total population and across different glycemic states were evaluated using receiver operating characteristic (ROC) curve analysis. The Akaike Information Criterion (AIC) and Bayesian Information Criterion (BIC) were calculated to compare the model fit for each IR-related marker. Models were adjusted for all covariates mentioned in the covariate section. These criteria were used to assess the predictive ability of each marker for heart disease and stroke across different glycemic states.

Multivariable-adjusted Cox regression models were used to explore the relationship between IR-related markers and the incidence of new-onset heart disease and stroke. Both continuous and quartile variables were used in the analysis to assess linear and nonlinear associations, and hazard ratios (HR) with 95% confidence intervals (CI) were calculated. Additionally, restricted cubic spline (RCS) analysis was performed to illustrate the dose-response relationship between IR-related markers and heart disease and stroke. The four knots in the RCS analysis were placed at the 5th, 35th, 65th, and 95th percentiles of the marker distributions. Both Cox analysis and RCS were conducted in the total population and across different glycemic states (normal, prediabetes, and diabetes).

Statistical analyses were performed using R version 4.2.0, with statistical significance set at P < 0.05 for all analyses.

## Results

### Participants’ characteristics

In this study, a total of 6009 participants were included, with an average age of 58.32 ± 8.53 years, and 55.9% were female. At baseline, 656 participants had heart disease and 108 had a stroke. During the 9-year follow-up period, there were 905 new cases of heart disease and 408 new cases of stroke. We found that the TyG, TyG-BMI, TyG-WC, TyG-WHtR, TG/HDL ratio, LAP index, and VAI showed significant differences among normal, prediabetic, and diabetic individuals (P < 0.001), with values increasing progressively. Conversely, the eGDR index also exhibited significant differences among the three groups but with decreasing values. Compared to individuals with normal glycemic levels, prediabetic and diabetic individuals tended to be older, have larger waist circumferences, higher BMIs, and higher values in the included blood parameters.

### Pearson correlation analysis of IR markers

**Figure 1:**
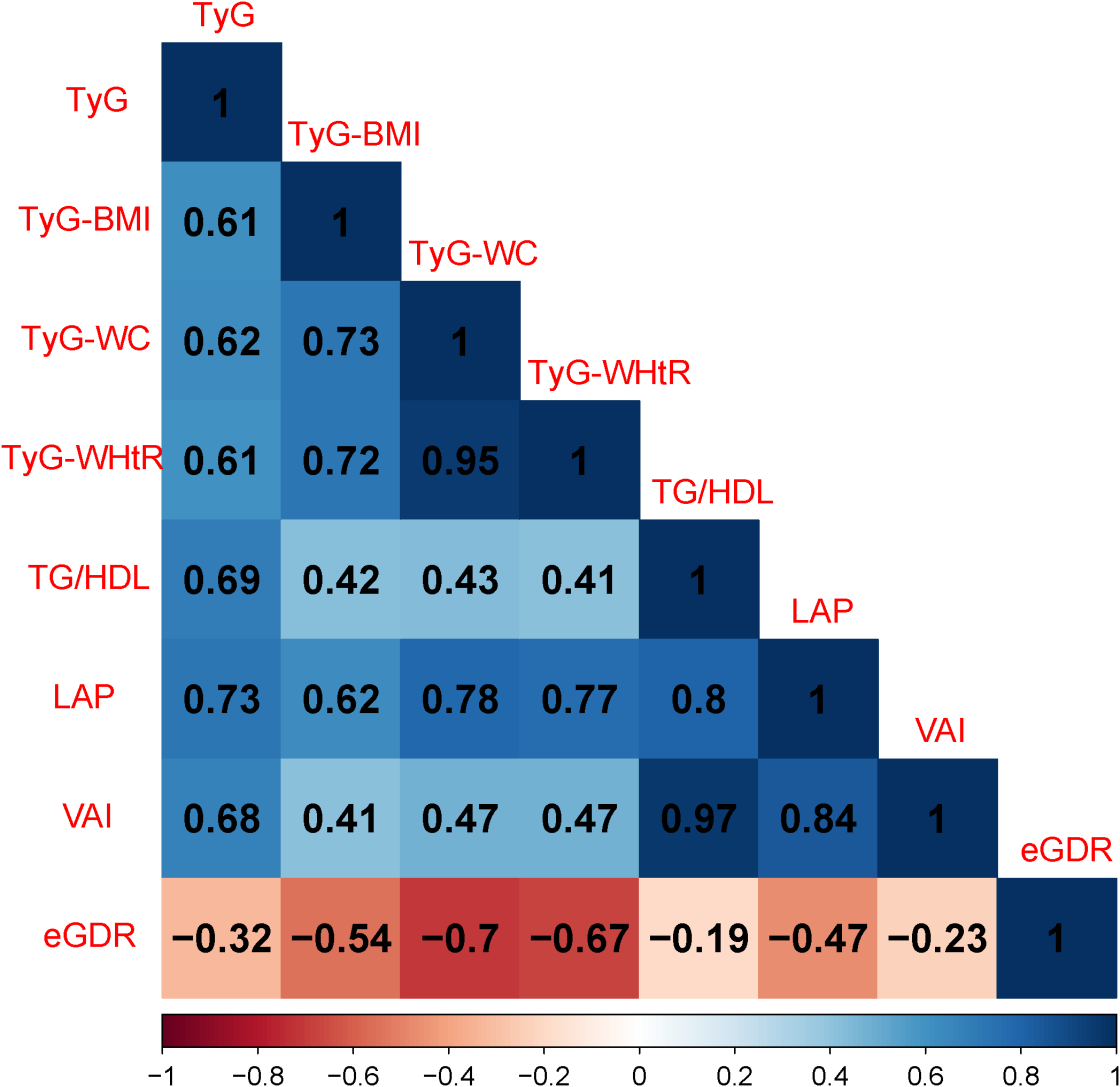
Pearson correlation analysis of IR-related markers. TyG, triglyceride-glucose index; BMI, body mass index; WC, waist circumference; WHtR, waist-to-height ratio; TG, triglyceride; HDL, high-density lipoprotein; LAP, lipid accumulation product; VAI, visceral adiposity index; eGDR, estimated glucose disposal rate.

TyG index showed moderate to strong positive correlations with all markers except eGDR index (r > 0.60). TyG-BMI index had high correlations with TyG-WC index (r = 0.73) and TyG-WHtR index (r = 0.72). Notably, the correlation coefficients between TyG-WC index and TyG-WHtR index (r = 0.95), as well as between TG/HDL ratio and VAI index (r = 0.97), were extremely high. TG/HDL index, VAI index, and LAP index also exhibited high positive correlations with each other (r ≥ 0.80). Additionally, eGDR index was negatively correlated with the other seven insulin resistance indices, with the strongest negative correlations observed with TyG-WC index (r = -0.70) and TyG-WHtR index (r = -0.67) (**Figure 1**).

### Predictive capacity comparison

For heart disease, the AUC values indicate that the eGDR index consistently has the highest predictive capacity across all glycemic states, with an overall AUC of 0.655. In normal and prediabetes, the eGDR index’s AUCs are 0.644 and 0.652, respectively. Conversely, the TyG index shows the lowest predictive capacity in the diabetes, with an AUC of 0.518.Among the IR-related markers, the TyG-WHtR index and LAP index follow, with overall AUCs of 0.629 and 0.615, respectively. For stroke, the eGDR index also exhibits the highest predictive capacity across all glycemic states, with an overall AUC of 0.716. In normal and prediabetes, the eGDR index’s AUCs are 0.737 and 0.668, respectively. The TyG index again shows the lowest predictive capacity in the diabetic group, with an AUC of 0.564 (**Table 2**).

**Table 2:** Characteristics of 6009 participants according to glycemic states.

Characteristics	Overall	Normal	Prediabetes	Diabetes	P
Number	6009	2415	2708	886	
Age (years)	58.32 (8.53)	57.53 (8.65)	58.74 (8.38)	59.21 (8.44)	<0.001
Sex (%)					0.984
Male	2649 (44.1)	1063 (44.0)	1197 (44.2)	389 (43.9)	
Female	3360 (55.9)	1352 (56.0)	1511 (55.8)	497 (56.1)	
Education (%)					0.453
illiteracy	1732 (28.8)	681 (28.2)	784 (29.0)	267 (30.1)	
Primary and below	3692 (61.4)	1480 (61.3)	1676 (61.9)	536 (60.5)	
Secondary and above	585 (9.7)	254 (10.5)	248 (9.2)	83 (9.4)	
Married statue (%)					0.897
Married	5193 (86.4)	2093 (86.7)	2335 (86.2)	765 (86.3)	
Other	816 (13.6)	322 (13.3)	373 (13.8)	121 (13.7)	
Smoking (%)					0.217
Yes	2219 (36.9)	921 (38.1)	969 (35.8)	329 (37.1)	
No	3790 (63.1)	1494 (61.9)	1739 (64.2)	557 (62.9)	
Drinking (%)					0.944
No	4064 (67.6)	1631 (67.5)	1837 (67.8)	596 (67.3)	
Yes	1945 (32.4)	784 (32.5)	871 (32.2)	290 (32.7)	
Hypertension (%)					<0.001
No	4554 (75.8)	1955 (81.0)	2050 (75.7)	549 (62.0)	
Yes	1455 (24.2)	460 (19.0)	658 (24.3)	337 (38.0)	
Kidney disease (%)					0.031
No	5633 (93.7)	2244 (92.9)	2563 (94.6)	826 (93.2)	
Yes	376 (6.3)	171 (7.1)	145 (5.4)	60 (6.8)	
Heart disease (%)					<0.001
No	5353 (89.1)	2189 (90.6)	2407 (88.9)	757 (85.4)	
Yes	656 (10.9)	226 (9.4)	301 (11.1)	129 (14.6)	
Stroke (%)					0.019
No	5901 (98.2)	2379 (98.5)	2662 (98.3)	860 (97.1)	
Yes	108 (1.8)	36 (1.5)	46 (1.7)	26 (2.9)	
BMI (Kg/m2)	23.67 (3.98)	23.00 (3.72)	23.84 (3.90)	24.98 (4.50)	<0.001
WC (cm)	84.47 (12.41)	82.29 (12.24)	85.17 (12.19)	88.29 (12.40)	<0.001
TyG index	8.67 (0.66)	8.37 (0.47)	8.72 (0.56)	9.31 (0.84)	<0.001
TyG-BMI index	205.85 (41.98)	192.93 (35.35)	208.41 (39.89)	233.22 (49.60)	<0.001
TyG-WC index	734.03 (134.61)	689.91 (117.23)	744.00 (127.15)	823.84 (149.61)	<0.001
TyG-WHtR index	4.66 (0.87)	4.38 (0.76)	4.72 (0.82)	5.23 (0.96)	<0.001
eGDR	9.83 (2.12)	10.35 (1.88)	9.79 (2.01)	8.52 (2.44)	<0.001
LAP index	37.71 (44.22)	26.79 (24.86)	38.57 (38.22)	64.81 (77.85)	<0.001
VAI index	2.35 (3.65)	1.65 (1.41)	2.31 (2.61)	4.40 (7.67)	<0.001
TG/HDL ratio	3.22 (4.84)	2.28 (1.75)	3.15 (3.30)	6.01 (10.33)	<0.001
Glucose (mg/dL)	108.55 (32.11)	91.57 (7.28)	108.02 (7.36)	156.45 (60.04)	<0.001
Hemoglobin (%)	5.27 (0.78)	5.00 (0.32)	5.22 (0.41)	6.17 (1.51)	<0.001
TC (mg/dL)	195.07 (38.60)	187.95 (34.95)	198.58 (38.65)	203.72 (44.32)	<0.001
Triglycerides (mg/dL)	131.54 (107.26)	105.50 (54.06)	132.84 (88.04)	198.55 (200.17)	<0.001
BUN (mg/dL)	15.64 (4.40)	15.33 (4.24)	15.81 (4.41)	15.98 (4.71)	<0.001
hsCRP (mg/dL)	2.45 (6.65)	2.00 (4.78)	2.58 (7.66)	3.27 (7.54)	<0.001
Uric acid (mg/dL)	4.38 (1.20)	4.25 (1.15)	4.45 (1.20)	4.49 (1.31)	<0.001
Creatinine (mg/dL)	0.77 (0.18)	0.76 (0.17)	0.77 (0.18)	0.78 (0.21)	0.012

Data are mean (SD), n (%), or median (IQR).

TyG, triglyceride-glucose index; BMI, body mass index; WC, waist circumference; WHtR, waist-to-height ratio; TG, triglyceride; HDL, high-density lipoprotein; LAP, lipid accumulation product; VAI, visceral adiposity index; eGDR, estimated glucose disposal rate; HbA1,: glycated hemoglobin; TC, total cholesterol; hsCRP, high-sensitivity C-reactive protein.

Comparing heart disease and stroke, it is evident that the eGDR index has a significantly higher predictive capacity for stroke (AUCs of 0.716 and 0.655, respectively). This trend is consistent across all glycemic states. Other IR-related markers such as TyG-WHtR, LAP index, TyG-BMI, TyG-WC, and VAIalso exhibit similar trends, showing higher predictive capacities for stroke than for heart disease (**Table 3**).

**Table 3:** Predictive capacity of insulin resistance-related markers for heart disease and stroke.

IR-related markers	Heart disease				Stroke			
	Overall	Normal	Prediabetes	Diabetes	Overall	Normal	Prediabetes	Diabetes
TyG index	0.567	0.566	0.558	0.518	0.605	0.567	0.603	0.564
TyG-BMI index	0.584	0.575	0.576	0.570	0.621	0.634	0.586	0.573
TyG-WC index	0.584	0.558	0.590	0.561	0.627	0.614	0.597	0.598
TyG-WHtR index	0.584	0.571	0.581	0.561	0.629	0.642	0.586	0.608
TG/HDL ratio	0.567	0.554	0.565	0.540	0.610	0.578	0.630	0.549
LAP index	0.585	0.574	0.583	0.555	0.615	0.611	0.596	0.585
VAI index	0.579	0.571	0.576	0.541	0.607	0.584	0.620	0.554
eGDR index	0.655	0.644	0.652	0.649	0.716	0.737	0.668	0.690

TyG, triglyceride-glucose index; BMI, body mass index; WC, waist circumference; WHtR, waist-to-height ratio; TG, triglyceride; HDL, high-density lipoprotein; LAP, lipid accumulation product; VAI, visceral adiposity index; eGDR, estimated glucose disposal rate; IR, insulin resistance.

**Figure 2:**
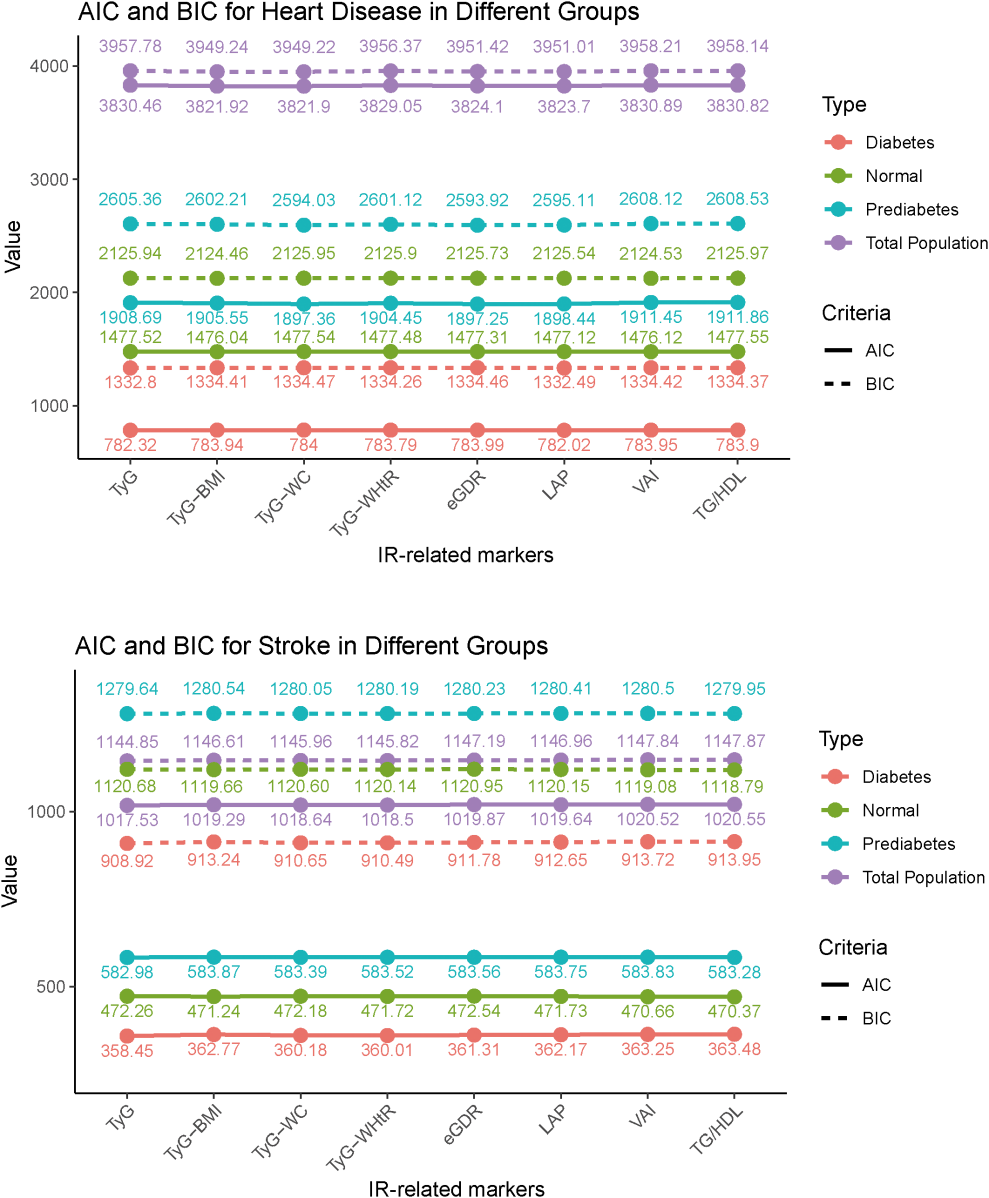
Akaike information criterion and Bayesian Information criterion analysis of heart disease and stroke prediction across IR-related markers. TyG, triglyceride-glucose index; BMI, body mass index; WC, waist circumference; WHtR, waist-to-height ratio; TG, triglyceride; HDL, high-density lipoprotein; LAP, lipid accumulation product; VAI, visceral adiposity index; eGDR, estimated glucose disposal rate.

The AIC and BIC results further support these findings. For heart disease, the TyG-WHtR index and LAP index show lower AIC and BIC values compared to the other markers. The TyG-BMI and TyG-WC also show notable predictive ability. In stroke, the eGDR index has the lowest AIC and BIC values across all glycemic states. Overall, the trends indicate that among the IR-related markers, the eGDR index, TyG-WHtR, and LAP index are more effective in predicting cardiovascular events, particularly with the eGDR index being especially strong in stroke prediction (**Figure 2**).

### Relationships between IR-related markers and cardiovascular disease in all participate

Each unit increase in the TyG, TyG-BMI, TyG-WC, and TyG-WHtR indices respectively increases the risk of heart disease by 25.1%, 0.4%, 0.1% and 17.6%, while the risk of stroke increases by 69.3%, 0.6%, 0.2% and 36.5%. Conversely, each unit increase in the eGDR index reduces the risk of heart disease and stroke by 21% and 14.2%, respectively (**Tables 4**, **5**).

**Table 4:** Associations of insulin resistance-related markers with heart disease.

IR-related markers	Overall		Normal		Prediabetes		Diabetes	
	HR (95%CI)	P	HR (95%CI)	P	HR (95%CI)	P	HR (95%CI)	P
TyG index (Continues)	1.251 (1.013,1.546)	3.76E-02	1.552 (0.825,2.92)	1.73E-01	1.244 (0.765,2.021)	3.79E-01	1.203 (0.855,1.694)	2.89E-01
Q1	Reference		Reference		Reference		Reference	
Q2	1.206 (1.991,1.467)	6.18E-02	1.330 (0.985,1.795)	6.25E-02	0.987 (0.717,1.358)	9.34E-01	1.257 (0.547,2.891	5.90E-01
Q3	1.175 (0.958,1.440)	1.22E-01	1.083 (0.698,1.681)	7.21E-01	1.194 (0.863,1.651)	2.84E-01	0.914 (0.404,2.067)	8.29E-01
Q4	1.217 (0.945,1.567)	1.28E-01	0.999 (0.431,2.315)	9.80E-02	1.085 (0.692,1.703)	7.22E-01	1.227 (0.571,2.637)	6.01E-01
TyG-BMI index (Continues)	1.004 (1.002,1.005)	6.02E-08	1.006 (1.003,1.008)	2.74E-05	1.003 (1.000,1.006)	6.49E-02	1.005 (1.002,1.007)	2.16E-04
Q1	Reference		Reference		Reference		Reference	
Q2	1.132 (0.922,1.390)	2.36E-01	1.138 (0.843,1.536)	3.99E-01	0.992 (0.725,1.358)	9.60E-01	2.340 (1.006,5.443)	4.83E-02
Q3	1.322 (1.075,1.624)	8.05E-03	1.629 (1.187,2.234)	2.51E-03	1.170 (0.859,1.594)	3.10E-01	1.515 (0.645,3.560)	3.40E-01
Q4	1.977 (1.507,2.339)	1.98E-08	2.350 (1.632,3.385)	4.45E-06	1.537 (1.093,2.160)	1.34E-02	3.067 (1.371,6.863)	6.38E-03
TyG-WC index (Continues)	1.001 (1.001,1.002)	3.44E-05	1.003 (1.001,1.004)	5.02E-05	1.000 (1.000,1.001)	3.41E-01	1.001 (1.000,1.003)	1.21E-02
Q1	Reference		Reference		Reference		Reference	
Q2	1.123 (0.914,1.379)	2.68E-01	1.066 (0.787,1.444)	6.81E-01	1.194 (0.879,1.623)	2.57E-01	1.237 (0.548,2.790)	6.09E-01
Q3	1.438 (1.178.1.756)	3.53E-04	1.665 (1.226,2.261)	1.09E-03	1.248 (0.919,1.694)	1.56E-01	1.878 (0.899,3.921)	9.35E-02
Q4	1.612 (1.290,2.010)	2.15E-05	2.441 (1.680,3.546)	2.80E-06	1.232 (0.876,1.734)	2.31E-01	2.111 (1.021,4.361)	4.37E-02
TyG-WHtR index (Continues)	1.176 (1.064,1.300)	1.45E-03	1.352 (1.117,1.636)	2.00E-03	1.038 (0.896,1.202)	6.21E-01	1.277 (1.027,1.589)	2.76E-02
Q1	Reference		Reference		Reference		Reference	
Q2	1.107 (0.902,1.358)	3.32E-01	1.163 (0.864,1.565)	3.19E-01	1.045 (0.767,1.424)	7.81E-01	1.461 (0.633,3.372)	3.75E-01
Q3	1.285 (1.049,1.573)	1.53E-02	1.436 (1.041,1.982)	2.77E-02	1.128 (0.834,1.525)	4.35E-01	1.813 (0.831,3.955)	1.35E-01
Q4	1.418 (1.311,1.777)	2.45E-03	2.105 (1.425,3.108)	1.83E-04	0.993 (0.703,1.403)	9.70E-01	2.241 (1.037,4.842)	4.01E-02
TG/HDL ratio (Continues)	0.988 (0.953,1.204)	4.95E-01	0.990 (0.838,1.171)	9.08E-01	1.010 (0.926,1.102)	8.16E-01	0.982 (0.935,1.032)	4.69E-01
Q1	Reference		Reference		Reference		Reference	
Q2	1.078 (0.883,1.316)	4.61E-01	0.973 (0.712,1.330)	8.64E-01	1.140 (0.839,1.549)	4.01E-01	1.266 (0.691,2.319)	4.46E-01
Q3	1.355 (1.112,1.652)	2.60E-03	1.359 (0.951,1.943)	9.19E-02	1.509 (1.106,2.059)	9.48E-03	0.982 (0.541,1.784)	9.53E-01
Q4	1.206 (0.941,1.547)	1.39E-01	1.106 (0.621,1.970)	7.32E-01	1.203 (0.782,1.848)	4.00E-01	1.343 (0.735,2.456)	3.78E-01
LAP index (Continues)	1.002 (0.999,1.005)	2.08E-01	1.012 (1.004,1.020)	2.73E-03	0.999 (0.995,1.003)	6.50E-01	1.001 (0.996,1.007)	6.20E-01
Q1	Reference		Reference		Reference		Reference	
Q2	1.174 (0.953,1.446)	1.31E-01	1.283 (0.938,1.756)	1.19E-01	1.050 (0.766,1.439)	7.63E-01	1.401 (0.721,2.722)	3.20E-01
Q3	1.527 (1.242,1.877)	5.81E-05	1.991 (1.418,2.795)	6.93E-05	1.276 (0.935,1.741)	1.24E-01	1.659 (0.877,3.139)	4.46E-01
Q4	1.498 (1.170,1.917)	1.33E-03	1.947 (1.195,3.173)	7.49E-03	1.162 (0.789,1.711)	4.47E-01	2.051 (1.084,3.882)	2.73E-02
VAI index (Continues)	0.980 (0.940,1.022)	3.55E-01	0.993 (0.826,1.193)	9.36E-01	0.976 (0.885,1.076)	6.21E-01	0.978 (0.927,1.033)	2.66E-01
Q1	Reference		Reference		Reference		Reference	
Q2	1.234 (1.007,1.514)	4.26E-02	1.378 (1.001,1.899)	4.96E-02	1.162 (0.853,1.583)	3.41E-01	1.032 (0.562,1.895)	9.18E-01
Q3	1.388 (1.125,1.713)	2.26E-03	1.780 (1.220,2.598)	2.78E-03	1.303 (0.939,1.807)	1.13E-01	0.832 (0.451,1.534)	5.56E-01
Q4	1.294 (1.000,1.676)	5.03E-02	1.425 (0.783,2.592)	2.43E-01	1.141 (0.839,1.549)	4.01E-01	1.258 (0.680,2.328)	4.64E-01
eGDR index (Continues)	0.923 (0.890,0.956)	1.18E-05	0.899 (0.846,0.955)	5.78E-04	0.973 (0.921,1.027)	3.19E-01	0.857 (0.789,0.930)	2.28E-04
Q1	Reference		Reference		Reference		Reference	
Q2	0.790 (0.660,0.945)	1.01E-02	0.767 (0.562,1.047)	9.44E-02	0.927 (0.711,1.208)	5.74E-01	0.564 (0.366,0.868)	9.20E-03
Q3	0.716 (0.578,0.887)	2.21E-03	0.668 (0.469,0.952)	2.57E-02	0.840 (0.613,1.151)	2.79E-01	0.504 (0.283,0.898)	2.01E-02
Q4	0.641 (0.515,0.708)	6.96E-05	0.573 (0.404,0.814)	1.86E-03	0.855 (0.617,1.184)	3.45E-01	0.296 (0.137,0.642)	2.06E-03

HR, hazard ratio; CI, confidence interval; TyG, triglyceride-glucose index; BMI, body mass index; WC, waist circumference; WHtR, waist-to-height ratio; TG, triglyceride; HDL, high-density lipoprotein; LAP, lipid accumulation product; VAI, visceral adiposity index; eGDR, estimated glucose disposal rate.

All factors were adjusted for age, sex, drinking, smoking, education, marital status, total cholesterol, triglycerides, creatinine, blood urea nitrogen, C-reactive protein, uric acid, diabetes, hypertension, and kidney disease. Besides, different glycemic states were not adjusted for diabetes.

**Table 5:** Associations between insulin resistance-related markers and stroke.

IR-related markers	Overall		Normal		Prediabetes		Diabetes	
	HR (95%CI)	P	HR (95%CI)	P	HR (95%CI)	P	HR (95%CI)	P
TyG index (Continues)	1.693 (1.254,2.286)	5.79E-04	5.634 (1.688,18.803)	4.93E-03	1.474 (0.818,2.655)	1.97E-01	1.442 (0.889,2.340)	1.38E-01
Q1	Reference		Reference		Reference		Reference	
Q2	1.587 (1.177,2.141)	2.45E-03	1.630 (1.028,2.584)	3.77E-07	1.670 (1.014,2.750)	4.40E-02	1.025 (0.305,3.444)	9.69E-01
Q3	1.662 (1.229,2.247)	9.61E-04	1.397 (0.704,2.776)	3.39E-01	1.862 (1.142,3.037)	1.27E-02	1.221 (0.407,3.664)	7.22E-01
Q4	1.379 (0.962,1.974)	7.99E-02	1.229 (0.324,4.669)	7.62E-02	1.152 (0.636,2.097)	6.35E-01	1.058 (0.364,3.073)	9.18E-01
TyG-BMI index (Continues)	1.006 (1.003,1.008)	3.90E-07	1.005 (1.001,1.010)	1.97E-02	1.006 (1.002,1.010)	8.34E-04	1.005 (1.001,1.009)	1.04E-02
Q1	Reference		Reference		Reference		Reference	
Q2	1.651 (1.205,2.261)	1.79E-03	2.572 (1.631,4.057)	4.81E-05	1.219 (0.748,1.986)	4.27E-01	0.711 (0.236,2.142)	5.44E-01
Q3	2.041 (1.493,2.789)	7.58E-06	2.573 (1.545,4.283)	2.79E-04	1.843 (1.168,2.906)	8.55E-03	1.021 (0.381,2.730)	9.68E-01
Q4	2.345 (1.674,3.286)	7.32E-07	2.884 (1.558,5.340)	7.51E-04	1.703 (1.029,2.817)	3.82E-02	1.950 (0.784,4.847)	1.51E-01
TyG-WC index (Continues)	1.002 (1.002,1.003)	3.59E-05	1.003 (1.001,1.005)	2.78E-03	1.002 (1.000,1.003)	2.95E-02	1.002 (1.000,1.004)	1.33E-01
Q1	Reference		Reference		Reference		Reference	
Q2	1.462 (1.065,2.009)	1.90E-02	1.554 (0.978,2.470)	6.19E-02	1.657 (1.023,2.686)	4.03E-02	0.609 (0.174,2.127)	4.37E-01
Q3	1.905 (1.401,2.589)	3.88E-05	2.255 (1.401,3.630)	8.17E-04	1.748 (1.081,2.825)	2.27E-02	1.698 (0.635,4.540)	2.91E-01
Q4	2.165 (1.558,3.009)	4.27E-06	3.255 (1.844,5.748)	4.72E-05	1.737 (1.043,2.893)	3.39E-02	1.710 (0.648,4.507)	2.79E-01
TyG-WHtR index (Continues)	1.365 (1.178,1.582)	3.42E-05	1.515 (1.127,2.037)	5.99E-03	1.253 (1.008,1.557)	4.18E-02	1.355 (0.996,1.843)	5.33E-02
Q1	Reference		Reference		Reference		Reference	
Q2	1.598 (1.171,2.182)	3.13E-03	2.022 (1.287,3.176)	2.25E-03	1.515 (0.944,2.432)	8.54E-02	0.724 (0.220,2.386)	5.96E-01
Q3	1.791 (1.312,2.446)	2.44E-04	2.119 (1.272,3.532)	2.25E-03	1.666 (1.047,2.649)	3.12E-02	1.343 (0.493,3.661)	5.64E-01
Q4	2.225 (1.587,3.121)	3.55E-06	3.523 (1.922,6.459)	4.66E-05	1.704 (1.022,2.839)	4.08E-02	1.678 (0.630,4.466)	3.00E-01
TG/HDL ratio (Continues)	0.985 (0.943,1.030)	5.15E-01	1.236 (0.980,1.558)	7.34E-02	0.998 (0.898,1.109)	9.75E-01	0.979 (0.914,1.050)	5.54E-01
Q1	Reference		Reference		Reference		Reference	
Q2	1.703 (1.254,2.312)	6.48E-04	2.401 (1.481,3.894)	3.84E-04	1.273 (0.803,2.020)	3.05E-01	2.343 (0.834,6.577)	1.06E-01
Q3	1.888 (1.388,2.567)	5.19E-05	2.150 (1.170,3.950)	1.37E-02	1.786 (1.148,2.777)	1.01E-02	2.421 (0.890,6.586)	8.34E-02
Q4	1.757 (1.227,2.516)	2.09E-03	3.618 (1.513,8.651)	3.84E-03	1.127 (0.648,1.959)	6.72E-01	2.505 (0.903,6.948)	7.76E-02
LAP index (Continues)	1.002 (0.998,1.006)	3.55E-01	1.007 (0.996,1.018)	2.36E-01	1.003 (0.997,1.009)	2.76E-01	0.998 (0.993,1.003)	4.38E-01
Q1	Reference		Reference		Reference		Reference	
Q2	1.726 (1.264,2.357)	5.99E-05	1.961 (1.219,3.154)	5.52E-03	1.580 (0.999,2.499)	5.03E-02	2.587 (0.824,8.117)	1.03E-01
Q3	1.918 (1.397,2.634)	5.59E-05	2.783 (1.635,4.737)	1.61E-04	1.380 (0.863,2.208)	1.79E-01	3.534 (1.185,10.537)	3.35E-02
Q4	2.402 (1.685,3.426)	1.30E-06	3.785 (1.822,7.863)	3.58E-04	1.921 (1.134,3.253)	1.52E-02	3.443 (1.147,10.340)	2.75E-02
VAI index (Continues)	0.984 (0.937,1.034)	5.29E-01	1.129 (0.861,1.480)	3.81E-01	0.981 (0.877,1.096)	7.32E-01	0.973 (0.903,1.048)	4.66E-01
Q1	Reference		Reference		Reference		Reference	
Q2	1.590 (1.170,2.161)	3.04E-03	2.205 (1.334,3.644)	2.03E-03	1.384 (0.886,2.163)	1.51E-01	2.401 (0.841,6.856)	1.02E-01
Q3	2.054 (1.505,2.803)	5.74E-06	3.895 (2.083,7.283)	2.06E-05	1.646 (1.043,2.599)	3.23E-02	2.823 (1.029,7.747)	4.39E-02
Q4	1.875 (1.291,2.723)	9.61E-04	7.076 (2.776,18.036)	4.16E-06	1.202 (0.685,2.110)	5.21E-01	2.463 (0.864,7.021)	9.17E-01
eGDR index (Continues)	0.858 (0.815,0.904)	8.98E-09	0.823 (0.752,0.900)	2.03E-05	0.892 (0.824,0.964)	4.18E-03	0.864 (0.770,0.970)	1.29E-02
Q1	Reference		Reference		Reference		Reference	
Q2	0.517 (0.380,0.705)	2.97E-05	0.617 (0.405,0.940)	2.47E-02	0.888 (0.626,1.259)	5.04E-01	0.580 (0.321,1.047)	7.09E-02
Q3	0.371 (0.265,0.520)	8.28E-09	0.429 (0.260,0.709)	9.58E-04	0.670 (0.428,1.048)	7.94E-02	0.338 (0.134,0.855)	2.19E-02
Q4	1.002 (0.998,1.006)	3.55E-01	0.272 (0.160,0.461)	1.33E-06	0.508 (0.306,0.841)	8.52E-03	0.398 (0.148,1.073)	6.87E-02

HR, hazard ratio; CI, confidence interval; TyG, triglyceride-glucose index; BMI, body mass index; WC, waist circumference; WHtR, waist-to-height ratio; TG, triglyceride; HDL, high-density lipoprotein; LAP, lipid accumulation product; VAI, visceral adiposity index; eGDR, estimated glucose disposal rate.

All factors were adjusted for age, sex, drinking, smoking, education, marital status, total cholesterol, triglycerides, creatinine, blood urea nitrogen, C-reactive protein, uric acid, diabetes, hypertension, and kidney disease. Besides, different glycemic states were not adjusted for diabetes.

**Figure 3:**
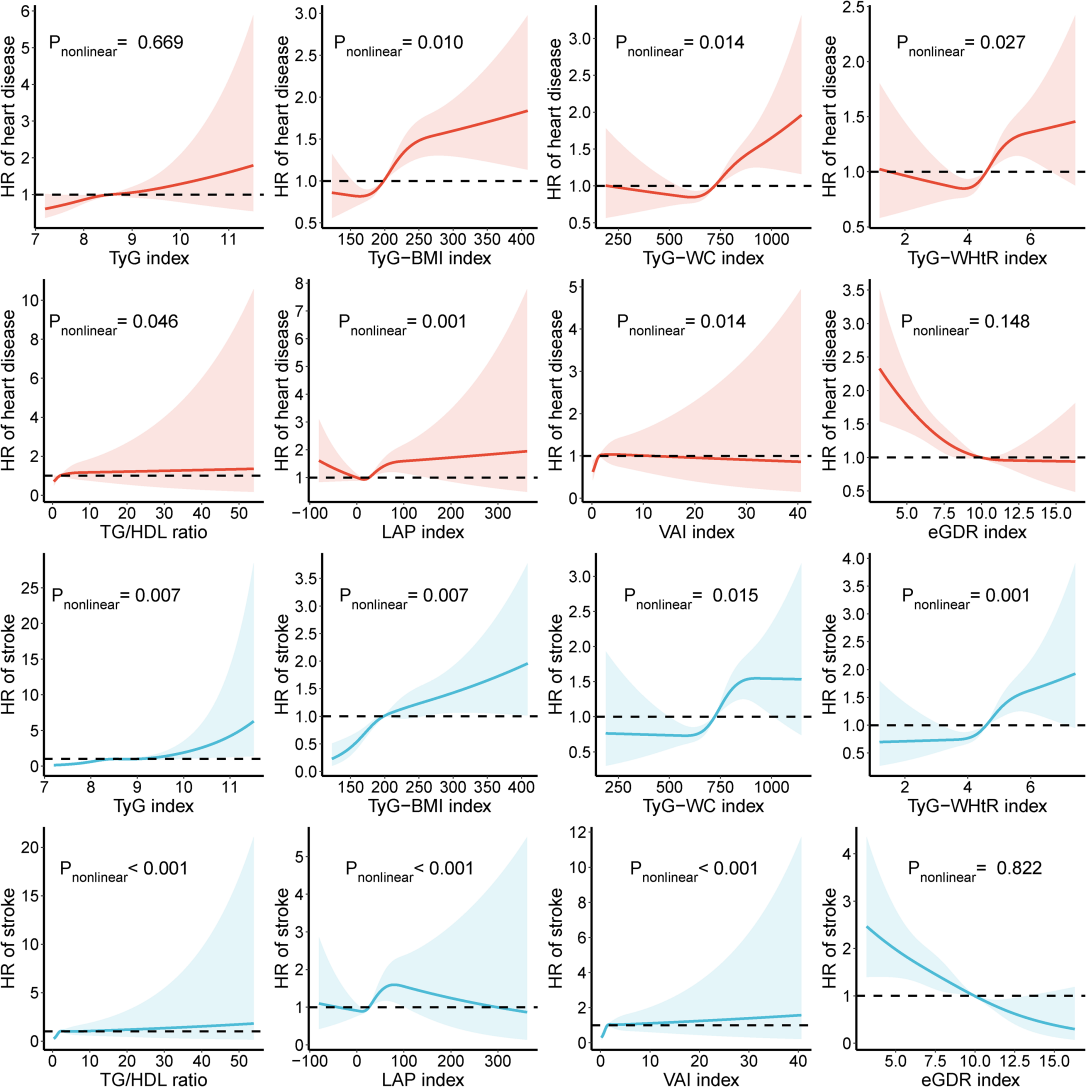
Association of IR-related markers with heart disease and stroke. HR, hazard ratio; CI, confidence interval; TyG, triglyceride-glucose index; BMI, body mass index; WC, waist circumference; WHtR, waist-to-height ratio; TG, triglyceride; HDL, high-density lipoprotein; LAP, lipid accumulation product; VAI, visceral adiposity index; eGDR, estimated glucose disposal rate. All factors were adjusted for age, sex, drinking, smoking, education, marital status, total cholesterol, triglycerides, creatinine, blood urea nitrogen, C-reactive protein, uric acid, hypertension, and kidney disease.

Compared to the Q1 group, higher levels (Q4) of TyG-BMI, TyG-WC, and TyG-WHtR indices increase the risk of heart disease by 97.7%, 61.2%, and 41.8%, respectively, and the risk of stroke by 2.345, 2.165, and 2.225 times, respectively. Compared to the Q1 group, TG/HDL, LAP, and VAI indices in the Q3 group have the highest risk of heart disease, increasing by 35.5%, 52.7%, and 38.8%, respectively. In the Q3 group, the risk of stroke associated with TyG, TG/HDL, and VAI indices is 1.662, 1.888, and 2.054 times that of the Q1 group. (**Tables 4**, **5**) The RCS results indicate that as the levels of TyG and TG/HDL increase, the risks of heart disease and stroke also rise, while eGDR shows an opposite trend. For heart disease risk, TyG-BMI, TyG-WC, TyG-WHtR, and LAP initially decrease and then increase, whereas VAI shows an initial increase followed by a decrease. Regarding stroke risk, as TyG-BMI and VAI increase, the risk gradually rises. TyG-WC and LAP exhibit a complex trend with stroke risk, initially decreasing, then increasing, and finally decreasing again (**Figure 3**).

### Relationships between IR-related markers and cardiovascular disease across different glycemic states

In individuals with normal glycemic levels, the risk of heart disease increases with the rise in TyG, TyG-BMI, TyG-WC, TyG-WHtR, LAP, and VAI indices. However, this relationship is not observed in prediabetic and diabetic individuals. Compared to the Q1 group, TyG-BMI, TyG-WC, TyG-WHtR, LAP, and VAI at Q2 and Q3 levels increase the risk of heart disease. TG/HDL increases heart disease risk in the Q3 group among prediabetic individuals. In individuals with normal glycemic levels and those with diabetes, eGDR at Q3 and Q4 levels significantly reduces the risk of heart disease (**Table 4**). Among individuals with normal glycemic levels, TyG-WC, LAP, and VAI exhibit a nonlinear association with heart disease, while only TyG-BMI and LAP show nonlinear associations in prediabetic and diabetic individuals, respectively (**Supplementary Figures 2-4**).

For stroke, high levels of IR-related indices, excluding eGDR, increase the risk of disease in individuals with normal glycemic levels. Among prediabetic individuals, high levels of TyG-BMI, TyG-WC, TyG-WHtR, and LAP also increase the risk of stroke. In diabetic individuals, only high levels of LAP are associated with an increased risk of stroke. (**Table 5**) Among individuals with normal glycemic levels, as the TyG-BMI value increases, stroke risk initially rises and then falls, while TyG-WC and TyG-WHtR show an initial decline followed by an increase. LAP presents an inverted “S” shaped curve with stroke risk, and the stroke risk gradually rises with higher TG/HDL and VAI indices. In prediabetic individuals, except for eGDR, other indices generally show a gradual rise in stroke risk. In diabetic individuals, TyG-WC and LAP show an initial rise followed by a decline in stroke risk. Across different glycemic states, eGDR consistently shows a negative correlation with stroke risk (**Supplementary Figures 2-4**).

## Discussion

In this study, we found that eGDR consistently demonstrated the highest predictive capacity for heart disease and stroke across all glycemic states. The TyG-WHtR and LAP indices also showed relatively high predictive capacities. Conversely, the TyG index had the lowest predictive capacity, particularly in the diabetic group. Additionally, eGDR, TyG-WHtR, and LAP were found to be independent risk factors for heart disease and stroke. These findings suggest that eGDR, TyG-WHtR, and LAP are more effective in predicting heart disease and stroke.

These IR-related indices reflect IR status from different dimensions, can be calculated using routine clinical data, and have good practicality and strong predictive value for CVD. The TyG index, which combines fasting glucose and triglycerides, is highly correlated with the HIEC, and is a cost-effective tool suitable for large-scale epidemiological studies (23). Its modified forms (such as TyG-WC, TyG-WHtR, and TyG-BMI) further incorporate measures of central obesity, enhancing the sensitivity for detecting insulin resistance across various metabolic states (24). The TG/HDL ratio is another well-established surrogate of IR and has been associated with both subclinical and clinical CVD. LAP and VAI integrate triglyceride levels with waist circumference or BMI, offering a dual view of dyslipidemia and adiposity. They have demonstrated superior CVD risk prediction compared to BMI alone, particularly among non-obese individuals and those with metabolic abnormalities (25, 26).

Previous studies have predominantly focused on either diabetic or non-diabetic populations when exploring the relationship between various TyG indices and CVD. Dang et al. (6) demonstrated that TyG-WC, TyG-WHtR and TyG-BMI could enhance the prediction of CVD. However, conflicting evidence exists, with some studies showing that TyG outperforms TyG-WHtR in predicting coronary heart disease (CHD) and stroke (20, 27). Other research suggests TyG-WC performs better in diabetic and prediabetic individuals, while TyG-WHtR may be more suitable for identifying early CVD risk in diabetic patients (28). We hypothesize that these inconsistencies may stem from variations in glycemic classifications. Our findings support the notion that TyG and its derivatives exhibit stronger predictive power in individuals with normal glycemic status, especially for stroke and heart disease.

Besides the TyG and its modified indices, other IR-related markers such as TG/HDL ratio, LAP, VAI, and eGDR index have also shown significant roles in predicting CVD. Similar to TyG, TG/HDL is also a reliable marker for assessing IR and is an independent risk factor for CVD, with a noted association with the severity of CVD (8). Recent studies have suggested a nonlinear relationship between TG/HDL and CVD risk. A UK Biobank study reported that while TG/HDL was positively associated with CVD, the risk increase slowed at higher levels, suggesting a saturation effect (8). Similarly, a Chinese cohort found that the risk rose sharply when TG/HDL exceeded 1.5, then plateaued (29). This nonlinear trend may be explained by the early impact of elevated TG/HDL on endothelial dysfunction, insulin resistance, and inflammation, which promote atherosclerosis. At higher levels, individuals may already have advanced metabolic impairment, where additional lipid changes contribute little to further CVD risk. Besides TG/HDL, the relationship of LAP and VAI with CVD and their predictive capacity for CVD across different glycemic states also warrant further discussion. Previous studies have shown that levels of TyG, TG/HDL, LAP, and VAI increase progressively from normal glucose levels to impaired fasting glucose, impaired glucose tolerance, and type 2 diabetes, with significant differences between groups (26). The Pearson correlation coefficients between these four IR-related markers and HIEC were found to be significant. However, when categorized by glycemic status, only LAP consistently demonstrated a significant association with IR across different glucose states. Additionally, the study indicated that LAP has the highest AUC for predicting CVD (26), consistent with our findings, where LAP exhibited the highest AUC among the four indicators for both heart disease and stroke. Nevertheless, compared to individual obesity markers like BMI, both LAP and VAI offer better predictive value for CVD (30). LAP and VAI also demonstrate good predictive performance for CVD in liver and kidney disease (31, 32). The relationships between LAP, VAI, and CVD risk exhibit a nonlinear pattern, suggesting that moderate levels of LAP and VAI may reflect metabolically functional subcutaneous or visceral adipose tissue. At this stage, fat depots retain sufficient storage capacity to safely sequester excess lipids and reduce ectopic fat accumulation in vital organs. This “metabolic buffering effect” may, to some extent, attenuate insulin resistance and systemic inflammation, thereby lowering the risk of cardiovascular events (33, 34). However, when these indices exceed a certain threshold, they may indicate excessive visceral fat accumulation and adipose tissue dysfunction, characterized by adipocyte hypertrophy, macrophage infiltration, and increased release of free fatty acids and pro-inflammatory cytokines. This transition from metabolic compensation to decompensation contributes to endothelial dysfunction, atherosclerosis, and increased risk of cardiovascular events (35).

Unlike other IR-related markers, as eGDR increases, the risk of CVD decreases progressively. The predictive value of eGDR for CVD is significant in both diabetic and non-diabetic patients (11). Compared to low levels of eGDR, higher levels of eGDR significantly reduce the risk of CVD. However, our study found that low levels of eGDR did not significantly increase the risk of heart disease, and another study using the CHARLS database did not reach this conclusion either (17). This discrepancy may be due to their selection of non-diabetic populations. Additionally, we did not observe a similar association in individuals with impaired glucose tolerance. For stroke, regardless of glycemic status, the risk decreases as eGDR levels drop. Further studies have shown that eGDR can reduce the risk of ischemic stroke but is not associated with hemorrhagic stroke. Overall, relative attributable risk shows that hypertension has the most significant impact on stroke (16, 36).

The modified TyG indices have demonstrated stronger predictive capabilities for both CVD and diabetes compared to the TyG index alone. TyG-WC and TyG-WHtR are particularly effective, with TyG-WC showing strong short-term predictive value for diabetes and TyG-WHtR excelling in long-term prediction (7). The TG/HDL ratio has a better ability to assess diabetes risk compared to single lipid markers (37). LAP, another key IR marker, is highly accurate in predicting diabetes, especially in middle-aged and elderly populations (38). VAI also effectively predicts diabetes across diverse populations, highlighting its broad applicability (39). Additionally, eGDR is effective not only for predicting type 2 diabetes but also shows strong predictive power for type 1 diabetes (16, 40).

These enhanced predictive abilities are likely due to the integration of variables such as BMI, WC, and height, which are closely linked to visceral fat accumulation—a key driver of IR through mechanisms like the secretion of pro-inflammatory cytokines, endothelial dysfunction, and atherosclerosis (41, 42). The modified TyG indices thus capture a broader spectrum of metabolic syndrome risk factors. On the other hand, eGDR’s strength in predicting CVD lies in its comprehensive reflection of metabolic function and IR, as it assesses glucose metabolism efficiency by integrating waist circumference, hypertension status, and HbA1c levels. The relationship between IR and hypertension further underlines eGDR’s effectiveness, as IR impacts blood pressure regulation through various mechanisms that ultimately increase the risk of cardiovascular events. HbA1c, as an indicator of long-term glycemic control, further contributes to eGDR’s predictive power by highlighting the long-term impact of poor glycemic management on endothelial function and atherosclerosis.

IR contributes to CVD through a series of complex mechanisms. Firstly, hyperinsulinemia resulting from IR promotes the proliferation and migration of vascular smooth muscle cells, which accelerates the development of atherosclerosis (43). Additionally, endothelial dysfunction is a key event in IR-related CVD, characterized by reduced nitric oxide (NO) bioavailability and increased endothelin-1 secretion, leading to an imbalance between vasodilation and vasoconstriction (44, 45). IR impairs insulin signaling, reducing endothelial nitric oxide synthase activation and NO production, while simultaneously increasing oxidative stress and reactive oxygen species levels, further depleting NO and causing direct endothelial cell damage (46). The presence of advanced glycation end-products in hyperglycemic conditions exacerbates this dysfunction by activating inflammatory and apoptotic pathways, leading to endothelial cell death and vascular stiffening. IR increases the secretion of pro-inflammatory cytokines such as TNF-α and IL-6, directly promoting chronic low-grade inflammation (47, 48). TNF-α disrupts insulin receptor signaling, perpetuating IR and inflammation, while IL-6 stimulates hepatic production of C-reactive protein, a key marker of systemic inflammation associated with elevated cardiovascular risk (49, 50). IR is strongly linked to lipid metabolism dysregulation, which further exacerbates CVD risk. IR increases hepatic production of very-low-density lipoproteins while reducing triglyceride-rich lipoprotein clearance, leading to hypertriglyceridemia. Meanwhile, lipoprotein lipase activity is suppressed, resulting in the accumulation of small, dense LDL particles that are highly atherogenic (51, 52). At the same time, HDL cholesterol levels decline, impairing reverse cholesterol transport and promoting lipid accumulation in arterial walls (53). Additionally, IR enhances oxidation of LDL, increasing foam cell formation and destabilizing atherosclerotic plaques. Furthermore, lifestyle interventions and pharmacological treatments play a key role in regulating IR and reducing CVD risk. Physical exercise enhances glucose uptake in skeletal muscle, improves insulin sensitivity, lowers circulating insulin levels, and alleviates IR-related metabolic disturbances (54, 55). Additionally, exercise has anti-inflammatory effects, modulating TNF-α and IL-6 secretion, reducing oxidative stress, and improving endothelial function, thereby lowering CVD risk (56). Pharmacological treatments also help improve IR and reduce CVD risk. Lipid-lowering medications (e.g., statins, fibrates) optimize lipid profiles, reduce vascular lipid deposition, and inhibit atherosclerosis progression (57). Glucose-lowering agents (e.g., metformin, SGLT2 inhibitors, GLP-1 receptor agonists) mitigate IR burden and lower CVD risk through mechanisms such as inhibiting hepatic gluconeogenesis, promoting glucose excretion, and improving vascular function (58).

However, our study did not take lifestyle factors, physical activity, or the use of glucose-lowering and lipid-lowering medications into account. Additionally, while eGDR demonstrated relatively stronger predictive value for CVD among the eight IR-related markers, and other indices like TyG and TG/HDL also showed good performance, we acknowledge that this does not indicate high predictive power in an absolute sense. These markers are only relatively better within the scope of our selected variables and were not directly compared with clinical gold standards such as the HEIC.

Nevertheless, these IR-related markers hold considerable potential for clinical application. They are derived from routine laboratory tests, easy to calculate, non-invasive, and cost-effective—making them suitable for the early detection of cardiometabolic risk, particularly among individuals without a formal diagnosis of diabetes. In clinical settings, they may serve as complementary tools to existing risk assessment models, especially in primary care and large-scale screening programs. Furthermore, integrating these metabolic indices with blood-based biomarkers, proteomic or genetic information, and lifestyle factors may enhance their predictive accuracy and support more personalized risk stratification in the future.

## Conclusion

The study concluded that eGDR consistently demonstrates the highest predictive capacity for heart disease and stroke across all glycemic states. In contrast, the TyG index and its modified forms, while useful, show relatively lower predictive values, especially in diabetic populations.

## Data Availability

The datasets presented in this study can be found in online repositories. The names of the repository/repositories and accession number(s) can be found below: https://charls.pku.edu.cn/.

## References

[1] Mensah GA Roth GA Fuster V. The global burden of cardiovascular diseases and risk factors: 2020 and beyond. J Am Coll Cardiol. (2019) 74:2529–32. doi: 10.1016/j.jacc.2019.doi: 10.009 31727292

[2] Vaduganathan M Mensah GA Turco JV Fuster V Roth GA. The global burden of cardiovascular diseases and risk: A compass for future health. J Am Coll Cardiol. (2022) 80:2361–71. doi: 10.1016/j.jacc.2022.11.005 36368511

[3] Ye Z Xu Y Tang L Wu M Wu B Zhu T. Predicting long-term prognosis after percutaneous coronary intervention in patients with new onset ST-elevation myocardial infarction: development and external validation of a nomogram model. Cardiovasc Diabetol. (2023) 22:87. doi: 10.1186/s12933-023-01820-9 37055777 PMC10103457

[4] Bonora E Targher G Alberiche M Bonadonna RC Saggiani F Zenere MB. Homeostasis model assessment closely mirrors the glucose clamp technique in the assessment of insulin sensitivity: studies in subjects with various degrees of glucose tolerance and insulin sensitivity. Diabetes Care. (2000) 23:57–63. doi: 10.2337/diacare.23.1.57 10857969

[5] Er LK Wu S Chou HH Hsu LA Teng MS Sun YC. Triglyceride glucose-body mass index is a simple and clinically useful surrogate marker for insulin resistance in nondiabetic individuals. PloS One. (2016) 11:e0149731. doi: 10.1371/journal.pone.0149731 26930652 PMC4773118

[6] Dang K Wang X Hu J Zhang Y Cheng L Qi X. The association between triglyceride-glucose index and its combination with obesity indicators and cardiovascular disease: NHANES 2003-2018. Cardiovasc Diabetol. (2024) 23:8. doi: 10.1186/s12933-023-02115-9 38184598 PMC10771672

[7] Kuang M Yang R Huang X Wang C Sheng G Xie G. Assessing temporal differences in the predictive power of baseline TyG-related parameters for future diabetes: an analysis using time-dependent receiver operating characteristics. J Transl Med. (2023) 21:299. doi: 10.1186/s12967-023-04159-7 37138277 PMC10158224

[8] Che B Zhong C Zhang R Pu L Zhao T Zhang Y. Triglyceride-glucose index and triglyceride to high-density lipoprotein cholesterol ratio as potential cardiovascular disease risk factors: an analysis of UK biobank data. Cardiovasc Diabetol. (2023) 22:34. doi: 10.1186/s12933-023-01762-2 36797706 PMC9936712

[9] Du T Yuan G Zhang M Zhou X Sun X Yu X. Clinical usefulness of lipid ratios, visceral adiposity indicators, and the triglycerides and glucose index as risk markers of insulin resistance. Cardiovasc Diabetol. (2014) 13:146. doi: 10.1186/s12933-014-0146-3 25326814 PMC4209231

[10] Qiao T Luo T Pei H Yimingniyazi B Aili D Aimudula A. Association between abdominal obesity indices and risk of cardiovascular events in Chinese populations with type 2 diabetes: a prospective cohort study. Cardiovasc Diabetol. (2022) 21:225. doi: 10.1186/s12933-022-01670-x 36320060 PMC9628026

[11] Zhang Z Zhao L Lu Y Xiao Y Zhou X. Insulin resistance assessed by estimated glucose disposal rate and risk of incident cardiovascular diseases among individuals without diabetes: findings from a nationwide, population based, prospective cohort study. Cardiovasc Diabetol. (2024) 23:194. doi: 10.1186/s12933-024-02256-5 38844981 PMC11157942

[12] Zhao S Wang Z Qing P Li M Liu Q Pang X. Comprehensive analysis of the association between triglyceride-glucose index and coronary artery disease severity across different glucose metabolism states: a large-scale cross-sectional study from an Asian cohort. Cardiovasc Diabetol. (2024) 23:251. doi: 10.1186/s12933-024-02355-3 39003471 PMC11245858

[13] Su J Li Z Huang M Wang Y Yang T Ma M. Triglyceride glucose index for the detection of the severity of coronary artery disease in different glucose metabolic states in patients with coronary heart disease: a RCSCD-TCM study in China. Cardiovasc Diabetol. (2022) 21:96. doi: 10.1186/s12933-022-01523-7 35668496 PMC9169264

[14] Ren Q Huang Y Liu Q Chu T Li G Wu Z. Association between triglyceride glucose-waist height ratio index and cardiovascular disease in middle-aged and older Chinese individuals: a nationwide cohort study. Cardiovasc Diabetol. (2024) 23:247. doi: 10.1186/s12933-024-02336-6 38992634 PMC11241990

[15] Wei X Min Y Song G Ye X Liu L. Association between triglyceride-glucose related indices with the all-cause and cause-specific mortality among the population with metabolic syndrome. Cardiovasc Diabetol. (2024) 23:134. doi: 10.1186/s12933-024-02215-0 38658993 PMC11044377

[16] Zabala A Darsalia V Lind M Svensson AM Franzen S Eliasson B. Estimated glucose disposal rate and risk of stroke and mortality in type 2 diabetes: a nationwide cohort study. Cardiovasc Diabetol. (2021) 20:202. doi: 10.1186/s12933-021-01394-4 34615525 PMC8495918

[17] Ren X Jiang M Han L Zheng X. Estimated glucose disposal rate and risk of cardiovascular disease: evidence from the China Health and Retirement Longitudinal Study. BMC Geriatr. (2022) 22:968. doi: 10.1186/s12877-022-03689-x 36517754 PMC9753298

[18] Zhao Y Hu Y Smith JP Strauss J Yang G. Cohort profile: the China health and retirement longitudinal study (CHARLS). Int J Epidemiol. (2014) 43:61–8. doi: 10.1093/ije/dys203 PMC3937970 23243115

[19] Li H Zheng D Li Z Wu Z Feng W Cao X. Association of depressive symptoms with incident cardiovascular diseases in middle-aged and older Chinese adults. JAMA Netw Open. (2019) 2:e1916591. doi: 10.1001/jamanetworkopen.2019.16591 31800066 PMC6902756

[20] Cui C Qi Y Song J Shang X Han T Han N. Comparison of triglyceride glucose index and modified triglyceride glucose indices in prediction of cardiovascular diseases in middle aged and older Chinese adults. Cardiovasc Diabetol. (2024) 23:185. doi: 10.1186/s12933-024-02278-z 38812015 PMC11138075

[21] Boris D Vanita RA George B Gretchen B Florence MB RaShaye F. Classification and diagnosis of diabetes: standards of medical care in diabetes-2022. Diabetes Care. (2022) 45:S17–s38. doi: 10.2337/dc22-S002 34964875

[22] Liang S Chen Y Sun X Dong X He G Pu Y. Long-term exposure to ambient ozone and cardiovascular diseases: Evidence from two national cohort studies in China. J Adv Res. (2024) 62:165–73. doi: 10.1016/j.jare.2023.08.010 PMC11331174 37625570

[23] Tahapary DL Pratisthita LB Fitri NA Marcella C Wafa S Kurniawan F. Challenges in the diagnosis of insulin resistance: Focusing on the role of HOMA-IR and Tryglyceride/glucose index. Diabetes Metab Syndr: Clin Res Rev. (2022) 16. doi: 10.1016/j.dsx.2022.102581 35939943

[24] Kuang M Yang R Huang X Wang C Sheng G Xie G. Assessing temporal differences in the predictive power of baseline TyG-related parameters for future diabetes: an analysis using time-dependent receiver operating characteristics. J Trans Med. (2023) 21. doi: 10.1186/s12967-023-04159-7 PMC10158224 37138277

[25] Qiao T Luo T Pei H Yimingniyazi B Aili D Aimudula A. Association between abdominal obesity indices and risk of cardiovascular events in Chinese populations with type 2 diabetes: a prospective cohort study. Cardiovasc Diabetol. (2022) 21. doi: 10.1186/s12933-022-01670-x PMC9628026 36320060

[26] Fiorentino TV Marini MA Succurro E Andreozzi F Sesti G. Relationships of surrogate indexes of insulin resistance with insulin sensitivity assessed by euglycemic hyperinsulinemic clamp and subclinical vascular damage. BMJ Open Diabetes Res Care. (2019) 7:e000911. doi: 10.1136/bmjdrc-2019-000911 PMC6861112 31798905

[27] Zheng S Shi S Ren X Han T Li Y Chen Y. Triglyceride glucose-waist circumference, a novel and effective predictor of diabetes in first-degree relatives of type 2 diabetes patients: cross-sectional and prospective cohort study. J Transl Med. (2016) 14:260. doi: 10.1186/s12967-016-1020-8 27604550 PMC5015232

[28] Zhang Y Wang F Tang J Shen L He J Chen Y. Association of triglyceride glucose-related parameters with all-cause mortality and cardiovascular disease in NAFLD patients: NHANES 1999-2018. Cardiovasc Diabetol. (2024) 23:262. doi: 10.1186/s12933-024-02354-4 39026233 PMC11264797

[29] Feng TY Chen C Sun G Zheng T. The nonlineard association between triglyceride to HDL cholesterol ratio and long-term heart disease risk: findings from China Health and Retirement Longitudinal Study (CHARLS). BMC Cardiovasc Disord. (2024) 24:639. doi: 10.1186/s12872-024-04308-w 39538136 PMC11562674

[30] Fakhrolmobasheri M Abhari AP Heidarpour M Paymannejad S Pourmahdi-Boroujeni M Saffari AS. Lipid accumulation product and visceral adiposity index for incidence of cardiovascular diseases and mortality; results from 13 years follow-up in Isfahan cohort study. Obes Sci Pract. (2024) 10:e713. doi: 10.1002/osp4.v10.1 38264005 PMC10804326

[31] Colantoni A Bucci T Cocomello N Angelico F Ettorre E Pastori D. Lipid-based insulin-resistance markers predict cardiovascular events in metabolic dysfunction associated steatotic liver disease. Cardiovasc Diabetol. (2024) 23:175. doi: 10.1186/s12933-024-02263-6 38769519 PMC11106932

[32] Chen HY Chiu YL Chuang YF Hsu SP Pai MF Yang JY. Visceral adiposity index and risks of cardiovascular events and mortality in prevalent hemodialysis patients. Cardiovasc Diabetol. (2014) 13:136. doi: 10.1186/s12933-014-0136-5 25280960 PMC4189758

[33] Virtue S Vidal-Puig A. Adipose tissue expandability, lipotoxicity and the Metabolic Syndrome–an allostatic perspective. Biochim Biophys Acta. (2010) 1801:338–49. doi: 10.1016/j.bbalip.2009.12.006 20056169

[34] Tchernof A Després JP. Pathophysiology of human visceral obesity: an update. Physiol Rev. (2013) 93:359–404. doi: 10.1152/physrev.00033.2011 23303913

[35] Ibrahim MM. Subcutaneous and visceral adipose tissue: structural and functional differences. Obes Rev. (2010) 11:11–8. doi: 10.1111/j.1467-789X.2009.00623.x 19656312

[36] Lu Z Xiong Y Feng X Yang K Gu H Zhao X. Insulin resistance estimated by estimated glucose disposal rate predicts outcomes in acute ischemic stroke patients. Cardiovasc Diabetol. (2023) 22:225. doi: 10.1186/s12933-023-01925-1 37633905 PMC10464388

[37] Yuge H Okada H Hamaguchi M Kurogi K Murata H Ito M. Triglycerides/HDL cholesterol ratio and type 2 diabetes incidence: Panasonic Cohort Study 10. Cardiovasc Diabetol. (2023) 22:308. doi: 10.1186/s12933-023-02046-5 37940952 PMC10634002

[38] Yu J Yi Q Hou L Chen G Shen Y Song Y. Transition of lipid accumulation product status and the risk of type 2 diabetes mellitus in middle-aged and older Chinese: A national cohort study. Front Endocrinol. (2021) 12:770200. doi: 10.3389/fendo.2021.770200 PMC8660859 34899605

[39] Amato MC Giordano C Galia M Criscimanna A Vitabile S Midiri M. Visceral Adiposity Index: a reliable indicator of visceral fat function associated with cardiometabolic risk. Diabetes Care. (2010) 33:920–2. doi: 10.2337/dc09-1825 PMC2845052 20067971

[40] Linn W Persson M Rathsman B Ludvigsson J Lind M Andersson Franko M. Estimated glucose disposal rate is associated with retinopathy and kidney disease in young people with type 1 diabetes: a nationwide observational study. Cardiovasc Diabetol. (2023) 22:61. doi: 10.1186/s12933-023-01791-x 36935526 PMC10024828

[41] Alizargar J Bai CH Hsieh NC Wu SV. Use of the triglyceride-glucose index (TyG) in cardiovascular disease patients. Cardiovasc Diabetol. (2020) 19:8. doi: 10.1186/s12933-019-0982-2 31941513 PMC6963998

[42] Ormazabal V Nair S Elfeky O Aguayo C Salomon C Zuñiga FA. Association between insulin resistance and the development of cardiovascular disease. Cardiovasc Diabetol. (2018) 17:122. doi: 10.1186/s12933-018-0762-4 30170598 PMC6119242

[43] Beneit N Fernández-García CE Martín-Ventura JL Perdomo L Escribano Ó Michel JB. Expression of insulin receptor (IR) A and B isoforms, IGF-IR, and IR/IGF-IR hybrid receptors in vascular smooth muscle cells and their role in cell migration in atherosclerosis. Cardiovasc Diabetol. (2016) 15:161. doi: 10.1186/s12933-016-0477-3 27905925 PMC5134076

[44] Shinozaki K Hirayama A Nishio Y Yoshida Y Ohtani T Okamura T. Coronary endothelial dysfunction in the insulin-resistant state is linked to abnormal pteridine metabolism and vascular oxidative stress. J Am Coll Cardiol. (2001) 38:1821–8. doi: 10.1016/S0735-1097(01)01659-X 11738280

[45] Sánchez A Martínez P Muñoz M Benedito S García-Sacristán A Hernández M. Endothelin-1 contributes to endothelial dysfunction and enhanced vasoconstriction through augmented superoxide production in penile arteries from insulin-resistant obese rats: role of ET(A) and ET(B) receptors. Br J Pharmacol. (2014) 171:5682–95. doi: 10.1111/bph.12870 PMC4290710 25091502

[46] Sansbury BE Hill BG. Regulation of obesity and insulin resistance by nitric oxide. Free Radic Biol Med. (2014) 73:383–99. doi: 10.1016/j.freeradbiomed.2014.05.016 PMC4112002 24878261

[47] Kahn SE Hull RL Utzschneider KM. Mechanisms linking obesity to insulin resistance and type 2 diabetes. Nature. (2006) 444:840–6. doi: 10.1038/nature05482 17167471

[48] Wellen KE Hotamisligil GS. Inflammation, stress, and diabetes. J Clin Invest. (2005) 115:1111–9. doi: 10.1172/JCI25102 PMC1087185 15864338

[49] Popa C Netea MG van Riel PL van der Meer JW Stalenhoef AF. The role of TNF-alpha in chronic inflammatory conditions, intermediary metabolism, and cardiovascular risk. J Lipid Res. (2007) 48:751–62. doi: 10.1194/jlr.R600021-JLR200 17202130

[50] Lainampetch J Panprathip P Phosat C Chumpathat N Prangthip P Soonthornworasiri N. Association of tumor necrosis factor alpha, interleukin 6, and C-reactive protein with the risk of developing type 2 diabetes: A retrospective cohort study of rural Thais. J Diabetes Res. (2019) 2019:9051929. doi: 10.1155/2019/9051929 31485456 PMC6702842

[51] Luciani L Pedrelli M Parini P. Modification of lipoprotein metabolism and function driving atherogenesis in diabetes. Atherosclerosis. (2024) 394:117545. doi: 10.1016/j.atherosclerosis.2024.117545 38688749

[52] Adiels M Olofsson SO Taskinen MR Borén J. Overproduction of very low-density lipoproteins is the hallmark of the dyslipidemia in the metabolic syndrome. Arterioscler Thromb Vasc Biol. (2008) 28:1225–36. doi: 10.1161/ATVBAHA.107.160192 18565848

[53] Elkanawati RY Sumiwi SA Levita J. Impact of lipids on insulin resistance: insights from human and animal studies. Drug Des Devel Ther. (2024) 18:3337–60. doi: 10.2147/DDDT.S468147 PMC11298177 39100221

[54] Lavie CJ Ozemek C Carbone S Katzmarzyk PT Blair SN. Sedentary behavior, exercise, and cardiovascular health. Circ Res. (2019) 124:799–815. 30817262 10.1161/CIRCRESAHA.118.312669

[55] Sampath Kumar A Maiya AG Shastry BA Vaishali K Ravishankar N Hazari A. Exercise and insulin resistance in type 2 diabetes mellitus: A systematic review and meta-analysis. Ann Phys Rehabil Med. (2019) 62:98–103. doi: 10.1016/j.rehab.2018.11.001 30553010

[56] Scheffer DDL Latini A. Exercise-induced immune system response: Anti-inflammatory status on peripheral and central organs. Biochim Biophys Acta Mol Basis Dis. (2020) 1866:165823. doi: 10.1016/j.bbadis.2020.165823 32360589 PMC7188661

[57] Marston NA Giugliano RP Im K Silverman MG O’Donoghue ML Wiviott SD. Association between triglyceride lowering and reduction of cardiovascular risk across multiple lipid-lowering therapeutic classes: A systematic review and meta-regression analysis of randomized controlled trials. Circulation. (2019) 140:1308–17. doi: 10.1161/CIRCULATIONAHA.119.041998 PMC6791781 31530008

[58] Preda A Montecucco F Carbone F Camici GG Lüscher TF Kraler S. SGLT2 inhibitors: from glucose-lowering to cardiovascular benefits. Cardiovasc Res. (2024) 120:443–60. doi: 10.1093/cvr/cvae047 PMC12001887 38456601

