# Supplementary material for: Association of insulin resistance-related indicators with cardiovascular disease in Chinese people with different glycemic states
Source: Front Endocrinol (Lausanne). 2025 Apr 17;16:1515559. doi: 10.3389/fendo.2025.1515559 (PMC12043448; doi:10.3389/fendo.2025.1515559)
Supplement: Supplementary file 1 [file Table1.docx]

Figure 1 Flow chart of patient recruitment.


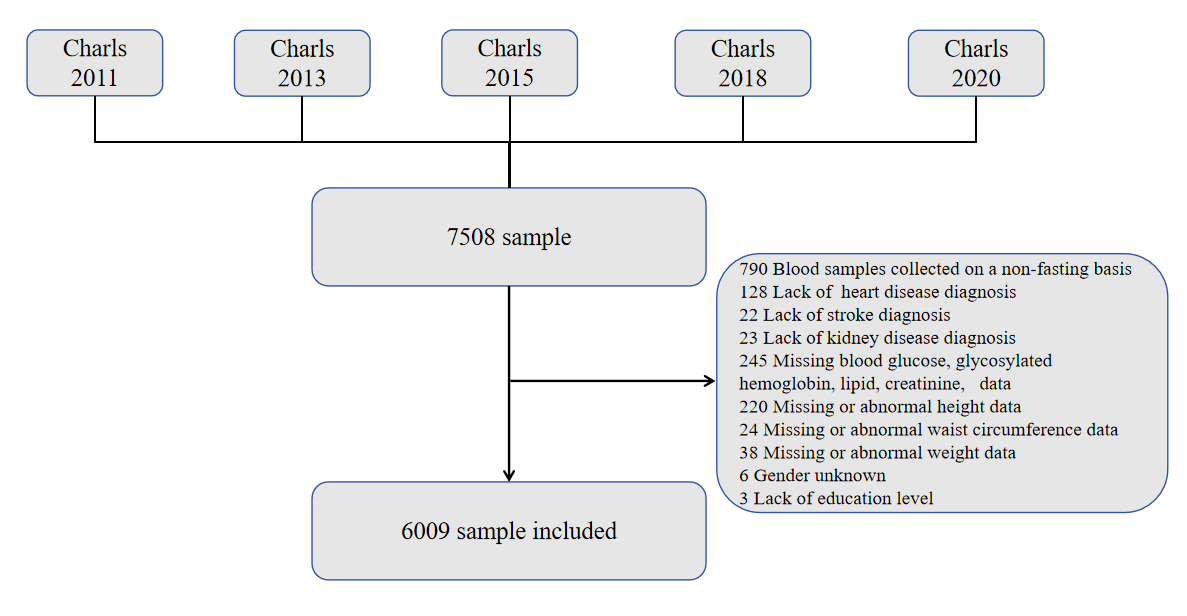


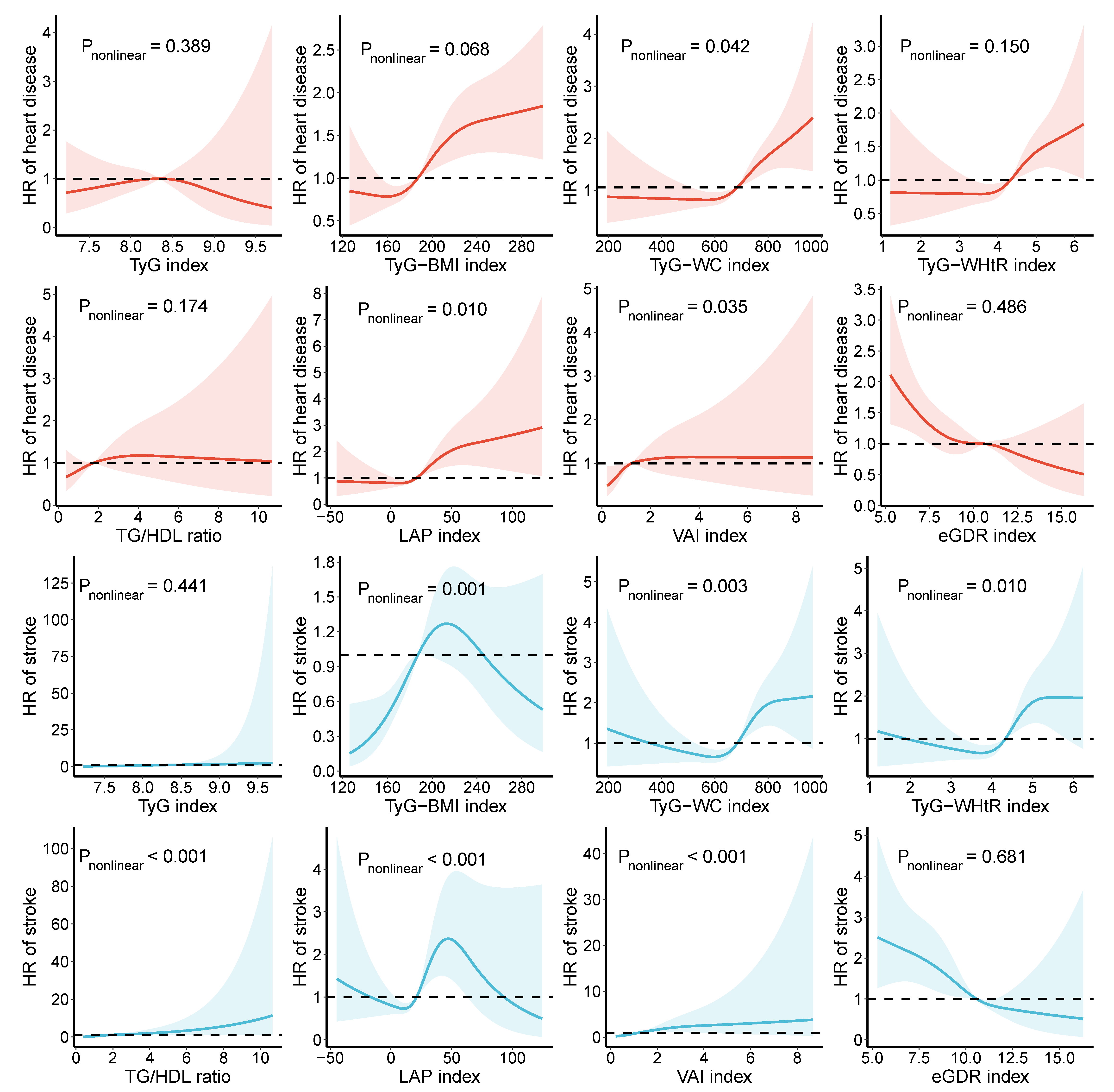
Figure 2 Association of IR-related index with heart disease and stroke in people with normal glycemic levels

Abbreviations: HR, hazard ratio; CI, confidence interval; TyG, triglyceride-glucose index; BMI, body mass index; WC, waist circumference; WHtR, waist-to-height ratio; TG, triglyceride; HDL, high-density lipoprotein; LAP, lipid accumulation product; VAI, visceral adiposity index; eGDR, estimated glucose disposal rate.

All factors were adjusted for age, sex, drinking, smoking, education, marital status, total cholesterol, triglycerides, creatinine, blood urea nitrogen, C-reactive protein, uric acid, hypertension, and kidney disease.


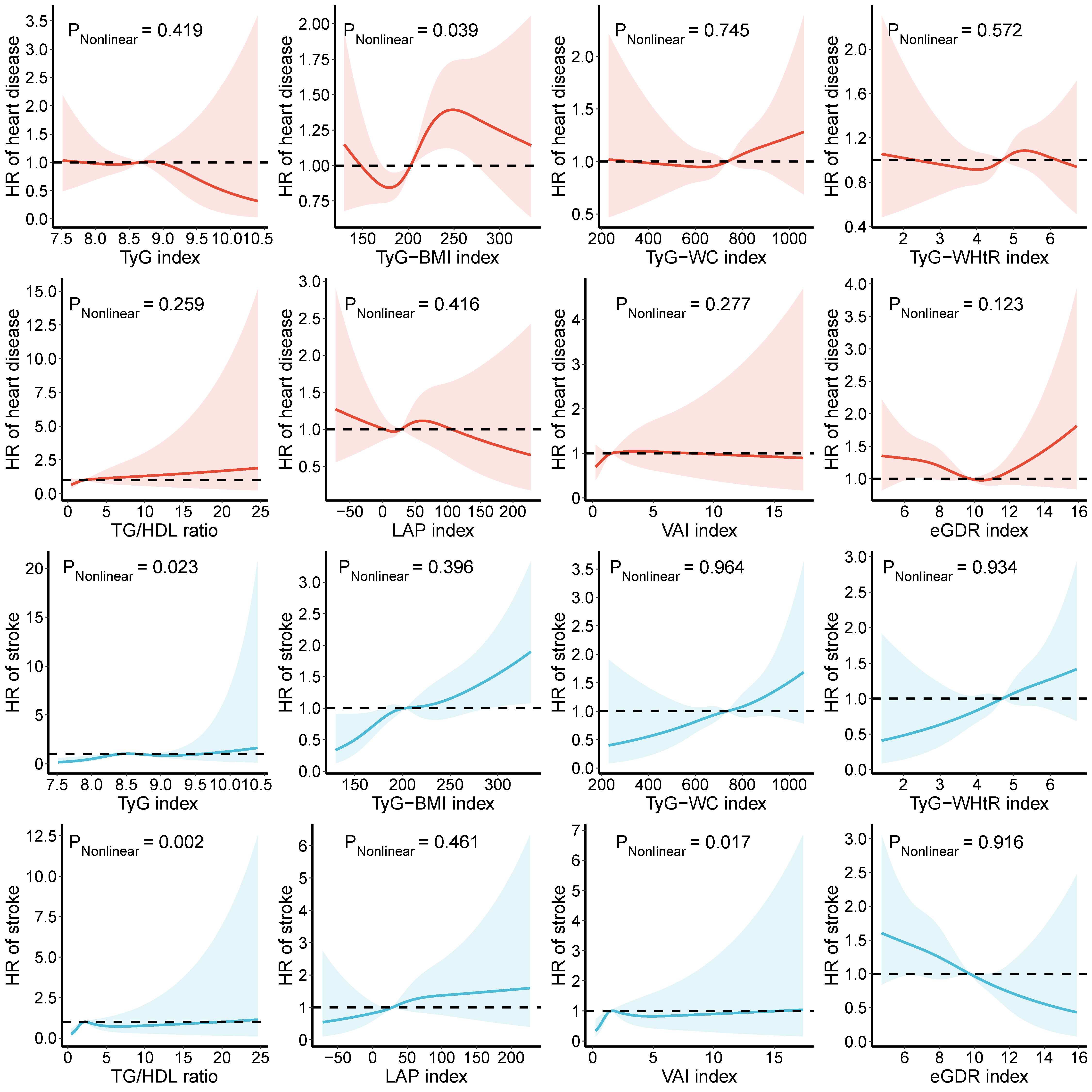
Figure 3 Association of IR-related index with heart disease and stroke in prediabetes people

Abbreviations: HR, hazard ratio; CI, confidence interval; TyG, triglyceride-glucose index; BMI, body mass index; WC, waist circumference; WHtR, waist-to-height ratio; TG, triglyceride; HDL, high-density lipoprotein; LAP, lipid accumulation product; VAI, visceral adiposity index; eGDR, estimated glucose disposal rate.

All factors were adjusted for age, sex, drinking, smoking, education, marital status, total cholesterol, triglycerides, creatinine, blood urea nitrogen, C-reactive protein, uric acid, hypertension, and kidney disease.


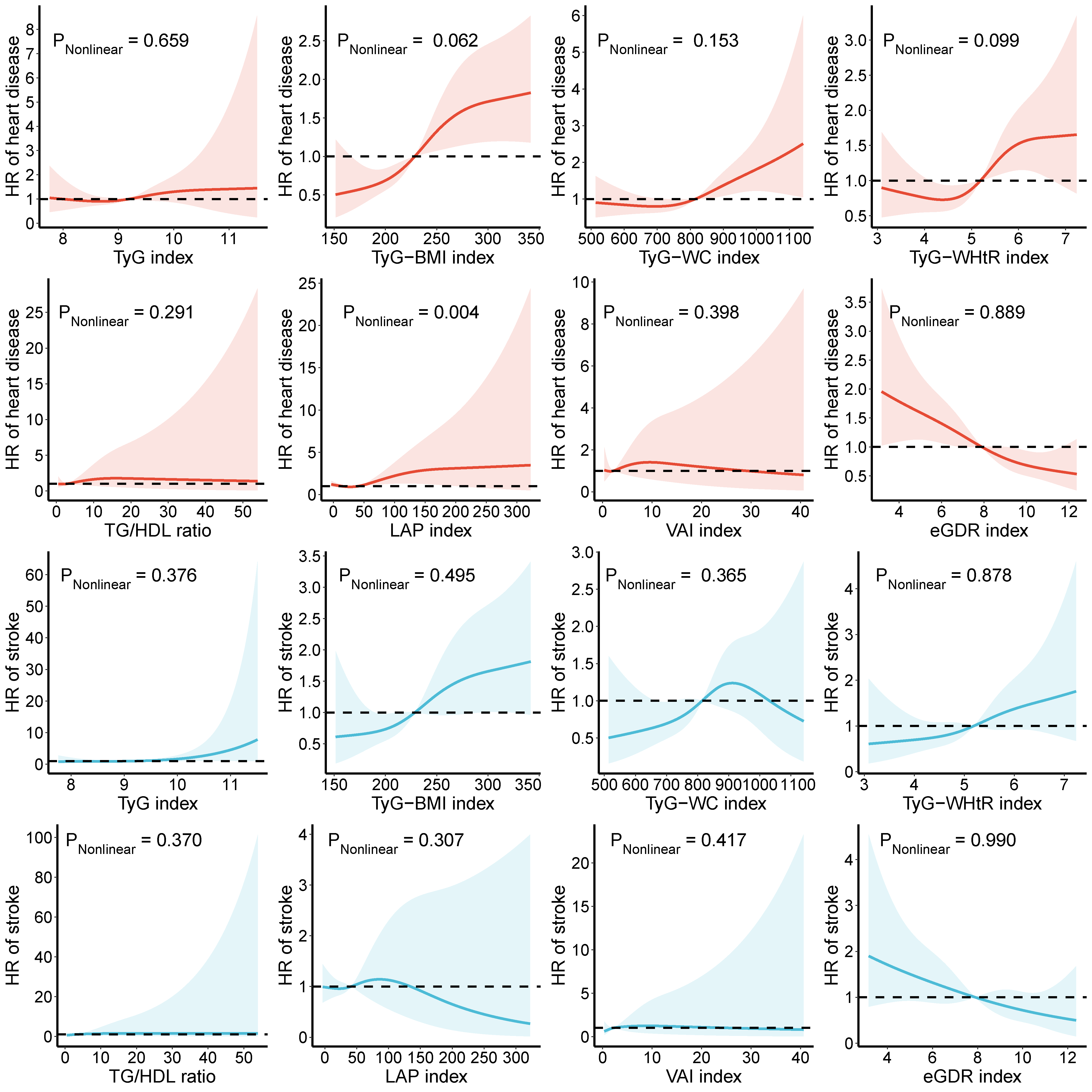
Figure 4 Association of IR-related index with heart disease and stroke in diabetes

Abbreviations: HR, hazard ratio; CI, confidence interval; TyG, triglyceride-glucose index; BMI, body mass index; WC, waist circumference; WHtR, waist-to-height ratio; TG, triglyceride; HDL, high-density lipoprotein; LAP, lipid accumulation product; VAI, visceral adiposity index; eGDR, estimated glucose disposal rate.

All factors were adjusted for age, sex, drinking, smoking, education, marital status, total cholesterol, triglycerides, creatinine, blood urea nitrogen, C-reactive protein, uric acid, hypertension, and kidney disease.
